# 3D-Printed Piezoelectret Based on Foamed Polylactic Acid for Energy-Harvesting and Sensing Applications

**DOI:** 10.3390/nano13222953

**Published:** 2023-11-15

**Authors:** Gabriele Perna, Francesco Bonacci, Silvia Caponi, Giacomo Clementi, Alessandro Di Michele, Luca Gammaitoni, Maurizio Mattarelli, Igor Neri, Debora Puglia, Francesco Cottone

**Affiliations:** 1 Department of Physics and Geology, University of Perugia, Via A. Pascoli, 06123 Perugia, Italy; gabriele.perna@studenti.unipg.it (G.P.); francesco.bonacci@unipg.it (F.B.); giacomo.clementi@unipg.it (G.C.); alessandro.dimichele@unipg.it (A.D.M.); luca.gammaitoni@unipg.it (L.G.); maurizio.mattarelli@unipg.it (M.M.); 2 Materials Foundry (IOM-CNR), National Research Council, c/o Department of Physics and Geology, Via A. Pascoli, 06123 Perugia, Italy; silvia.caponi@cnr.it; 3 Department of Civil and Environmental Engineering, University of Perugia, Strada di Pentima 4, 05100 Terni, Italy; debora.puglia@unipg.it

**Keywords:** innovative materials, cellular polymers, piezoelectrets, 3D printing, energy harvesting, polylactic acid

## Abstract

Poly(lactic) acid (PLA) is a bio-compatible polymer widely used in additive manufacturing, and in the form of cellular foam it shows excellent mechanical and piezoelectric properties. This type of structure can be easily 3D-printed by Fusion Deposition Modelling (FDM) with commercially available composite filaments. In this work, we present mechanical and electrical investigations on 3D-printed low-cost and eco-friendly foamed PLA. The cellular microstructure and the foaming degree were tuned by varying extrusion temperature and flowrate. The maximum surface potential and charge stability of disk samples were found in correspondence of extrusion temperature between 230 and 240 °C with a flowrate of 53–44% when charging on a heated bed at 85 °C. The cells’ morphology and correlated mechanical properties were analyzed and the measured piezoelectric $d_{33}$ coefficient was found to be 212 pC/N. These findings show the importance of printing parameters and thermal treatment during the charging process in order to obtain the highest charge storage, stability and material flexibility. These results suggest that 3D-printed cellular PLA is a promising sustainable material for sensing and energy-harvesting applications.

## 1. Introduction

Piezoelectrets are electroactive materials with an electrically charged cellular structure that can convert mechanical energy into electric energy and vice versa [1,2,3,4]. These materials, consisting of a solid polymer matrix containing a gas phase, are usually used in smart devices to measure or generate electrical signals, e.g., in mechanical, electro-acoustic and ultrasonic sensors, actuators and energy harvesters [5,6,7,8]. Differently from classical piezoelectric materials, for which piezoelectricity is due to the presence of electric dipole moments in solids with an asymmetric crystalline structure, cellular polymers can present piezoelectric activity through internal charging by subjecting the material to a high external electric field, thereby through an engineered poling process [9,10]. They are mainly based on polymers like polypropylene (PP), polyethylene (PE), polyethylene terephthalate (PET), fluorinated ethylene propylene (FEP) or polytetrafluoroethylene (PTFE), and some of these show sizable electromechanical and piezoelectric *d*${}_{33}$ coefficients together with flexibility, easy handling and low cost [7,11]. However, the major drawback of such materials is the fact that they are carbon-based and fabricated from fossil fuels, coupled to a poor recycling ability of discarded devices. Therefore, they do not comply with the eco-friendly policies necessary to reduce the negative impact on the environment [12].

Polylactic acid (PLA) is a biodegradable and bioabsorbable polymer originating from renewable plant sources like corn, potato starch and sugar cane. It can degrade after use in CO${}_{2}$ and water to re-enter natural circulation, thus representing a sustainable choice for energy conversion and sensor applications [13,14], and it is the most widely used plastic filament material in additive manufacturing. PLA in the form of cellular foams shows excellent mechanical and piezoelectric properties (*d*${}_{33}≃596$ pC/N [13], *d*${}_{33}≃539$ pC/N [14], *d*${}_{33}≃320$ pC/N [15]). It is now easily possible to implement this type of structure by Fusion Deposition Modelling (FDM) with commercially available composite filaments.

In this work, we show mechanical and electrical investigations on 3D-printed low-cost and eco-friendly foamed PLA material. In particular, several samples of Light-Weight PLA (LW-PLA) from the ColorFabb company were constructed. The cellular microstructure and the foaming morphology were tuned by varying the nozzle extrusion temperature and the flowrate. Disk-shaped samples were polarized using a negative corona discharge setup [16,17,18,19] on a pre-heated bed plate with an optimized thermal treatment. Morphological, thermal and mechanical analyses were performed on the material in order to correlate the elastic properties to the porosity and charge stability. Optimal charging conditions were found and charge stability was analyzed.

## 2. Material and Instrumental Setup

The material used for the fabrication of the samples is a modified expanding PLA commercial filament (ColorFabb LW-PLA natural HBP60C), using an active foaming technology to achieve light weight and low density through the presence of an expanding agent (a saturated hydrocarbon in ≤2.5 wt%). The material has a density range of 1.21–1.43 g/cm${}^{3}$ (when it is not activated) and a maximum activated density of 0.40–0.47 g/cm${}^{3}$ (when it is foamed), a glass transition temperature of 55–60 °C and the nominal diameter of the filament is 2.85 ± 0.10 mm. In order to preliminarily investigate the behaviour of the spool material and its blowing agent under temperature, a differential scanning calorimeter (DSC) and thermogravimetric analysis (TGA) were conducted. A fragment of the filament was heated from 25 °C to 180 °C with a heating rate of 10 °C/min (first heating scan), then cooled to −40 °C and finally heated again to 180 °C (second heating scan) at the same heating rate, for the DSC analysis, while its thermal decomposition was examined under nitrogen atmosphere in a temperature range of 25–600 °C with a heating rate of 10 °C/min.

The samples were then printed in a disk shape of 10 mm of nominal diameter and 0.5 mm of nominal thickness, through an Ultimaker3 FDM 3D printer. The fixed printing parameters were extrusion speed, layer height, build plate temperature and infill and were set to 70 mm/s, 0.1 mm, 25 °C and 100%. The foaming of the polymer, due to the expansion of the blowing agent inside the material, was tuned during printing choosing the only two variable parameters, i.e., the nozzle extrusion temperature and the flowrate. The extrusion temperature was set from 190 °C to 260 °C with steps of 10 °C (from 200 °C to 210 °C with steps of 5 °C) to gradually investigate the formation and nucleation of polymer cells. The flow of the material extruded from the nozzle was adjusted, as shown in Table 1, to compensate the expansion of the deposited filament due to the blowing agent evaporation. In particular, the material flowrate was set in order to maintain constant perimeter wall thickness and thus volume [20].

**Figure 1:**
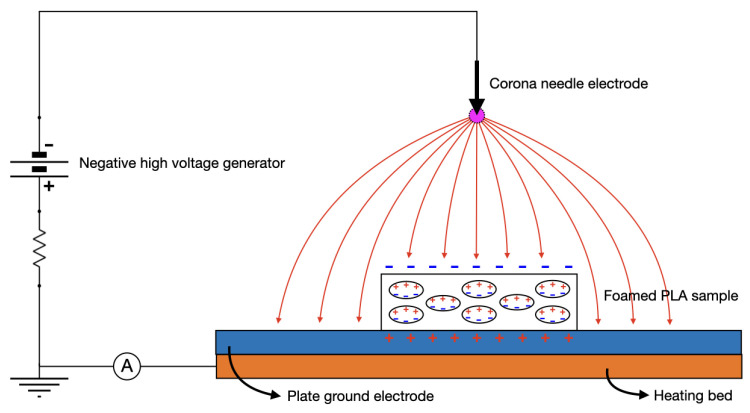
Schematic diagram of experimental negative corona charging setup on the build plate.

In order to charge the material and induce negative and positive charges to the inner surfaces of polymer cavities, the foamed LW-PLA samples were polarized first at room temperature (RT), then at a chosen pre-heating temperature T${}_{p}$, in a negative corona charging setup, as shown in Figure 1. They were placed on the 3D printer heated plate used as ground electrode of the high voltage generator. The needle-plate distance and pre-heating temperature have been varied to find the optimal ones. Finally, the surface potential of the samples was measured by an electrostatic voltmeter (Eltex Electric Field Meter EM03) while placed at a fixed distance of 2 cm from the sample, and the temporal decay of the potential was recorded up to a maximum of 5 min.

The mechanical characterization for all specimens has been carried out using the universal testing machine LRX-Plus by Lloyd Instruments Ltd., Bognor Regis, UK with a XLC series load cell of 2.5 kN. The data were recorded and analyzed using the computer software Nexygen MT v4.6.2 by Lloyd Instruments. Cylinders with nominal diameter of 10 mm and nominal height of 20 mm were printed at different temperatures and thus with different flowrate and foaming factor. Their mechanical properties have been investigated by compressing the samples up to a maximum load of 500 N along the height direction. The Young’s modulus, *E*, of the samples was estimated in compliance with BS2782: Part 3: Method 345A and ISO 604 [21,22]. Correlative Raman–Brillouin measurements have been carried out by a homemade set-up recently built in the GHOST Lab in Perugia (Italy) [23] in order to obtain the real and imaginary parts of the longitudinal elastic modulus of the 3D-printed PLA at different extrusion temperatures. The laser was focused at the surface of the samples, making the effect of multiple scattering present in optically inhomogeneous materials negligible [24]. The Raman measurements also provided details on the possible changes in molecular composition.

The data for investigating the piezoelectric effect of the electret samples and calculating the piezoelectric coefficient were acquired through a low-noise current preamplifier (Stanford Research Systems SR570, Sunnyvale, CA, USA) coupled with a digital oscilloscope (TBS 1152B by Tektronix Inc., Beaverton, OR, USA). Charged disks with 2 cm diameter and 500 μm thickness were compressed with a maximum force of 10 N and with a compression speed of 10 mm/s.

## 3. Results and Discussion

### 3.1. Material Characterization

**Figure 2:**
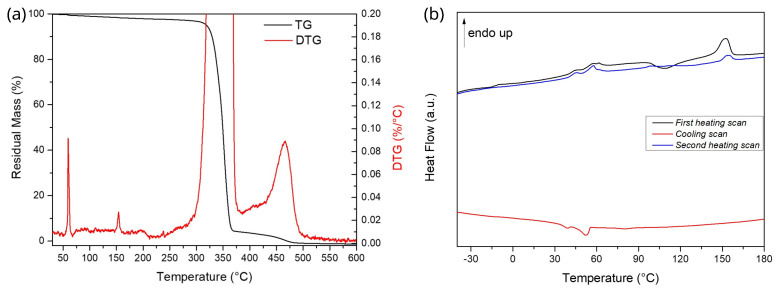
(**a**) Thermogravimetric and (**b**) differential scanning calorimeter analysis of spool material.

The analysis of DSC curves shows a glass transition temperature ($T_{g}$) at 58.09 °C, a cold crystallization temperature ($T_{cc}$) at 108.18 °C and a melting temperature ($T_{m}$) at 152.40 °C. At these experimental conditions, PLA was able to crystallize from the melt only at quite low temperatures during cooling. In parallel to the melting of the prevalent polymeric phase, the presence of a low-melting polymeric additive, i.e., the blowing agent, having a melting peak at 45.30 °C is registered, as shown in Figure 2. The nature of the compound is unknown, but the foaming activity of LW-PLA starting at nearly 205 °C can be due to the thermal degradation of the additive itself. Both melting events were also registered in TGA tests as sharp peaks in DTG curves.

### 3.2. Morphological Characterization

**Figure 3:**
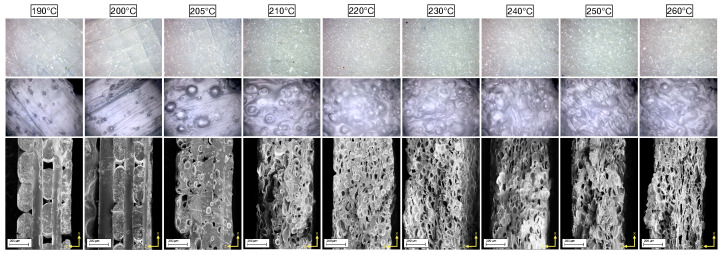
Different zoom of optical images of 3D-printed LW-PLA samples and relative SEM images of their cross sections.

In order to investigate the morphology of the piezoelectret samples, the spool material was 3D-printed with different nozzle extrusion temperatures: 190, 200, 205, 210, 220, 230, 240, 250 and 260 °C. Figure 3 shows optical images of the samples at different zoom level and SEM images of their cross sections. At low temperatures of 190, 200 and 205 °C, the disks are quite transparent and few bubbles are present inside, while above 210 °C the samples start to foam, become granular and the deposited layers tend to expand.

**Figure 4:**
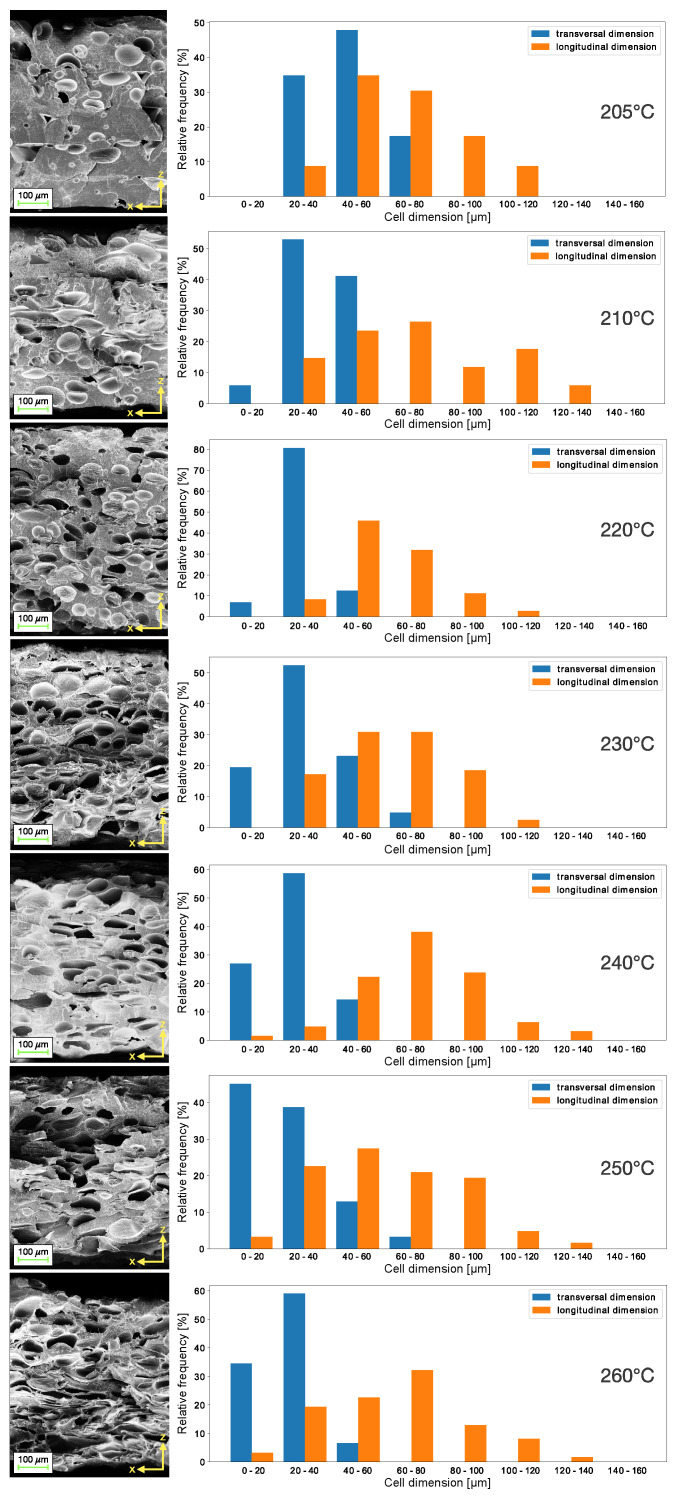
SEM images of the samples’ cross sections under various foaming temperatures with respective cell transversal and longitudinal dimensions histograms.

To obtain scanning electron microscope (SEM) images of the internal structure of the samples, the disks were then cut in a liquid nitrogen bath and coated by a conductive metal through sputtering. As confirmed by SEM images in Figure 3, bottom row, the sample layers extruded at 190 and 200 °C do not still adhere completely, leaving large longitudinal and transversal voids, following the direction of printing. From the temperature of 205 °C, for which the five composing layers are still visible, the microspheres of the blowing agent mixed in the PLA matrix start to evaporate, creating the bubbles nuclei. Since the pressure of the gas inside the cell is inversely proportional to the radius of the cell, the bubbles will grow in size, expanding their diameter and volume, as a result of nucleation [25]. Above the temperature of 210 °C, the layered structure is no longer observed and the cell number increases with temperature, reaching its maximum at 230 °C, at which the bubbles are maximum in size. Increasing the extrusion temperature, the cell shape tends to modify, becoming flatter and longer, showing the maximum effect at 260 °C. This can be observed in cell dimension histograms reported in Figure 4, along with SEM images. For low temperatures, the transversal dimension of the bubbles amounts between 25 and 50 μm, and the longitudinal dimension is around 50 μm, while, for high temperatures, the transversal dimension shifts to a range between 5 and 25 μm and the longitudinal dimension shifts to around 75 μm. The morphological change in the material is attributed to two factors: first, when the extrusion temperature exceeds 250 °C, the material starts to degrade and becomes less viscous as observed regarding falling as a result of gravity, squeezing the bubbles inside; second, as the temperature increases, the material remains viscous for longer before it solidifies, so the bottom layers cannot sustain the increasing weight of the layers printed on top of them, resulting in a slightly inhomogeneous morphology.

This behaviour is reflected in sample thickness and cell density trends as functions of extrusion temperature, as shown in Table 2. The thickness reaches its maximum value at 230 °C, which represents the optimal foaming temperature of the material, and then it decreases slightly at higher temperatures below the values obtained at 190/200 °C as the material changes morphology. By analyzing the SEM images of the samples’ cross sections illustrated in Figure 4, the surface cell density was computed by fitting the cavity boundaries to ellipses using the software ImageJ v1.53t. The values of cell density increase almost linearly with temperature, reaching a pleateau at 230 °C, where they then remain stable. The change in cell morphology can also be demonstrated by extracting from the cell dimension data the asphericity parameter $A=1-4ab/{\left(a+b\right)}^{2}$, where *a* and *b* are, respectively, the major and minor axes of the ellipses fitted over bubble shapes.

**Figure 5:**
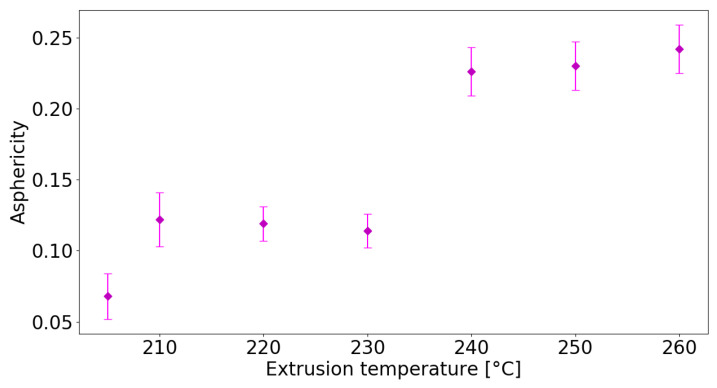
Average asphericity of sample cells printed at different extrusion temperatures.

Asphericity measures the deviation from a spherical shape and A∼0 when bubbles are spherical (a=b), while A∼1 when they have very elongated shapes (a≫b). Figure 5 shows that the smallest asphericity values (A≈0.1), corresponding to the roundest cell shape, are found for the samples printed at temperatures lower than 230 °C, where the most pronounced foaming effect is observed. Then, *A* raises rapidly at 240 °C and stabilizes to values close to A≈0.25, which correspond to the most squeezed shape for the internal cells.

**Figure 6:**
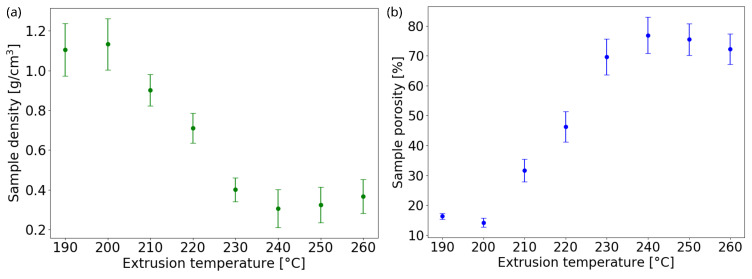
Calculated sample (**a**) density and (**b**) porosity as functions of nozzle extrusion temperature.

In Figure 6a, the sample density $\rho_{\mathrm{cellular}}$ is presented, measured from the mass and volume of the printed disks, versus extrusion temperature. It starts from a value of 1.104 g/cm${}^{3}$ for the smallest temperature and reaches a minimum of 0.305 g/cm${}^{3}$ at *T* = 230–240 °C, i.e., almost a quarter of the density of the bulk PLA. Then, the value rises mildly again because, at higher temperatures, the internal cells squeeze, reducing the foaming effect. The porosity degree, $P=1-\rho_{\mathrm{cellular}}/\rho_{\mathrm{solid}}$, is estimated from the ratio of foamed sample density (extruded from 190 °C to 260 °C) to the solid sample density (assumed as bulk PLA density 1.32 g/cm${}^{3}$). The foaming and bubble squeezing effects are reflected on porosity, as shown in Figure 6b. Starting from P≈16% at 190 °C, the porosity reaches its maximum of 77% at 240 °C, confirming the range of optimal foaming temperatures, and then decreases slightly again to a value of P≈72% at 260 °C.

### 3.3. Mechanical Characterization

**Figure 7:**
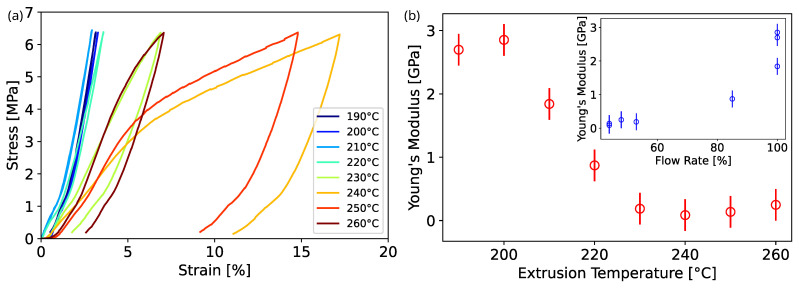
Mechanical characterization of PLA 3D-printed cylinders: (**a**) compressive stress as a function of percentage compressive strain; (**b**) measured Young’s modulus as a function of extrusion temperature; (inset) Young’s modulus representation with respect to flowrate percentage.

In Figure 7, the results of the mechanical analysis of the samples under compressive stress are presented, showing substantial differences as a function of temperature and foaming degree (i.e., material density). Figure 7a presents typical stress–strain curves for samples printed at different temperatures, where a distinctive degree of deformation is attained in terms of maximum compressive strain for a fixed maximum stress. Samples with a higher degree of foaming show plastic deformation evidenced by a hysteresis loop where the unloading cycle ends with the sample permanently strained. Figure 7b presents the corresponding Young’s modulus extracted from the linear region of the stress–strain curve. The inset of Figure 7b shows *E* as a function of printing flowrate percentage. At low temperatures, below 205 °C, the foaming effect is negligible and a constant Young’s modulus of around 2.7 GPa is observed, in accordance with typical values obtained on printed PLA [26,27]. Upon increasing the temperature and thus reducing the material density, a decrease in the Young’s modulus is found, which reaches a minimum of about 100 MPa for the samples printed at 240 °C with a flowrate of 44%.

The extrusion temperature also affects the strength of the material, reducing its elastic region [28], as shown Figure 7a. Meanwhile, the samples printed at low temperature, hence with higher Young’s modulus, maintain an elastic behaviour up to 500 N, and the plastic region appears at lower loads for the samples printed at higher temperatures and lower density.

**Figure 8:**
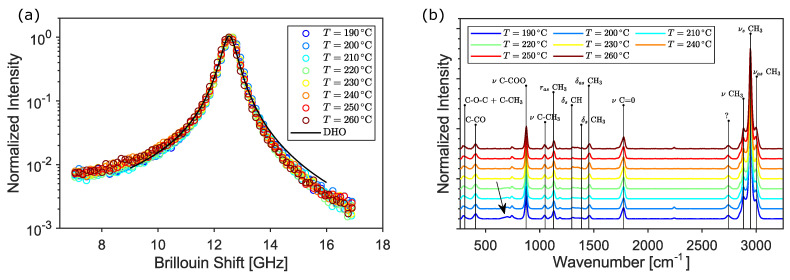
Brillouin (**a**) and Raman (**b**) spectra acquired at different printing temperatures.

To investigate whether the origin of the mechanical properties variation is due to the foaming structure or to a degradation of the material due to the extrusion temperature, simultaneous Raman–Brillouin measurements were conducted on the printed disks in back-scattering geometry. In fact, Raman and Brillouin spectra only depend on the microscopic structure and are unaffected by the relatively large voids present in the samples [29]. Typical Brillouin spectra at different extrusion temperatures are reported in Figure 8a. Data were fitted using the theoretical expression expected for a viscoelastic material (DHO function, solid black line):

(1) $$I\left(\omega \right)=\frac{I_{0}}{\pi }\frac{\omega_{B}^{2}\Gamma }{{\left(\omega^{2}-\omega_{B}^{2}\right)}^{2}+\omega^{2}\Gamma^{2}},$$

where $\omega_{B}$ and Γ are the position and the width of the Brillouin peak, respectively. A temperature-independent frequency shift of $\omega_{B}=12.56\pm 0.03$ GHz (mean ± std) was obtained. The Brillouin peak $\omega_{B}$ is linearly linked to the exchanged wavevector $q_{bs}$ through the longitudinal acoustic velocity in the medium, $\omega_{B}=q_{bs}v_{L}$, which in turn is related to the real part of the longitudinal elastic modulus $M^{\prime }=\rho v_{L}^{2}$. Considering the density of the PLA filament (ρ=1.32 g/cm${}^{3}$), the obtained data yield a constant modulus of $M^{\prime }=6.8$ GPa corresponding to a Young’s modulus of E=4.1 GPa, given ν=0.36 as the typical Poisson ratio of bulk PLA [30]. From the peak width Γ, which is proportional to the imaginary part of the elastic modulus of the material $M^{\prime \prime }=\omega_{B}\Gamma \rho /q_{bs}^{2}$, it is possible to evaluate the loss tangent $M^{\prime \prime }/M^{\prime }$ of 3D-printed PLA, which takes the value of 0.01. As illustrated by the Brillouin tests, no significant temperature-induced variation in the PLA elastic modulus is observed; indeed, the fluctuations are within the range of repeatability of the Brillouin measurement when performed at different points of the same sample.

The findings in this work are corroborated by performing a Raman study of the printed samples. Figure 8b shows the Raman spectra at different temperatures along with the most prominent characteristic peaks of PLA (black vertical lines). The peaks are assigned according to the deformation modes of the respective molecules: $\nu_{s}$ and $\nu_{as}$ stem, respectively, for the symmetric and asymmetric stretching vibrations; $\delta_{s}$ and $\delta_{as}$ similarly indicate symmetric and asymmetric deformation modes, while *r* indicates a rocking vibration [31]. Overall, the molecular structure of the PLA is preserved at increasing temperatures, indicating that the material is not degrading. Only a tiny difference is observable at small wavenumbers, where a broad and shallow peak at around 700 cm${}^{-1}$ (black arrow), found for the lower-temperature samples, progressively disappears when increasing the extrusion temperature.

**Figure 9:**
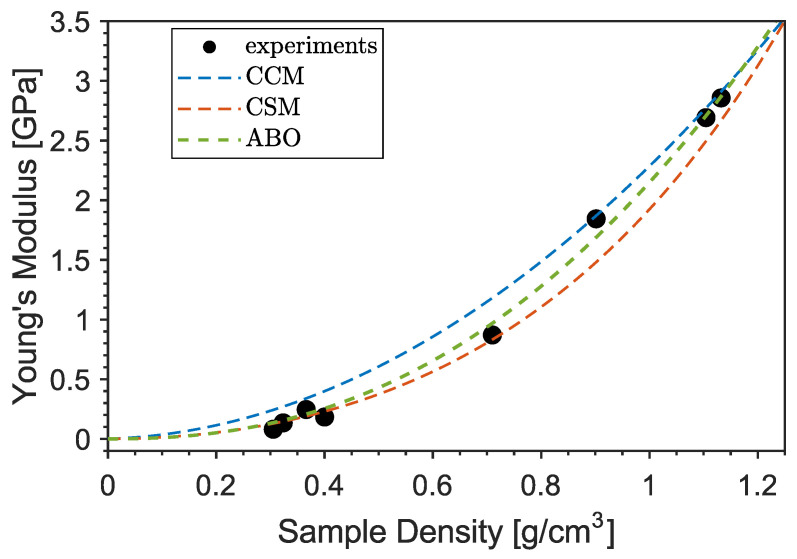
Young’s modulus versus density of the 3D-printed cylinders.

The origin and gradual decrease in this peak may be related to the evaporation of the unknown blowing agent occurring at temperatures higher than 200 ${}^{{}^{\circ}}$C, in agreement with the results of thermoanalysis. The combined Brillouin–Raman measurements indicate the absence of temperature-induced effects in the mechanical properties of bulk PLA. Therefore, the decrease in Young’s modulus of the 3D-printed samples has to be attributed only to the corresponding change in morphology and density due to the foaming effect. This is shown in Figure 9, where the Young’s modulus of the printed cylinders is plotted as a function of their density (black symbols). The obtained data were tested against typical non-linear models for the elastic modulus of foamed materials, such as the Cubic Cell Model [32] (CCM, blue line), the Composite Sphere Model of Ramakrishnan and Arunachalam [33] (CSM, orange) and the calculation of Arnold and co-authors [34] (ABO, green) based on oblate spheroidal inclusions of axis ratio a/b, with the rotational axis *a* oriented perpendicular to the stress direction, as suggested by the previous SEM analysis. The fitting function and the relative parameters are reported in Table 3 using $\rho_{\mathrm{solid}}=1.32$ g/cm${}^{3}$ and $E_{s}=4.1$ GPa for the density and Young’s modulus (extrapolated from Brillouin measurements) of the bulk PLA, respectively. In the Cubic Cell Model, the contribution of the gas pressure inside the pores is neglected; the only free parameter is the volume fraction of solid ϕ contained in the cell edges. A value of ϕ=0.95 is obtained, which is a reasonable value considering that the pores are surrounded by rather thick walls of matrix material, as evidenced by the SEM images. However, a cubic unit cell is not representative of the observed microstructure as the inclusions are almost spherical. In the Composite Sphere Model, the microstructure is approximated as an assembly of composite spheres of different sizes, each of which is composed of a central core and a shell representing, respectively, the inclusion and the matrix material. The only adjustable parameter is the Poisson ratio at zero porosity $\nu_{0}$. A good agreement with experiments can be found if the value of bulk PLA $\nu_{0}=0.36$ is used [30], as shown by the orange curve in Figure 9. In order to take into account the squeezed shape of the pores and their orientation with respect to the stress direction, the model of Arnold and co-authors [34] for spheroidal inclusions was also tested. A direct fit (green line) using a constant axis ratio as an adjustable parameter yields a value of a/b≈7, which is larger than the typical value measured by SEM. This may be related to the 2-dimensional nature of the SEM analysis, which probably underestimates the true aspect ratio of the pores. In conclusion, it is found that data can be well captured by rather approximated models that neglect the complex microstructure of the samples and assume simple geometrical pore shapes. Although a full mechanical characterization of PLA-based foamed materials was not the main scope of this work, we want to point out that a larger number of constitutive and semi-empirical models with increased complexity can be found in the literature [30].

### 3.4. Charging Methods and Electrical Behaviour

Foamed LW-PLA disks printed at the optimal extrusion temperature of 230 °C with 47.23% of porosity were preliminary polarized at room temperature (RT) of 25 °C by varying the needle-plate distance on the charging setup from 1 cm to 5 cm, immediately measuring the potential induced on the sample surface through the electrometer. The generator voltage was chosen in such a way as to avoid electric discharge in the instrumental setup. The selected voltage values are represented in Table 4, with the respective needle-plate distances and the calculated corresponding electric field values on sample surface, according to the needle-to-plate electrostatic model [35].

(2) $$E\left(x\right)=\frac{2V}{\left(r_{w}+2x\right)\ln\left(2L/r_{w}+1\right)}$$

In this model, the generator voltage is applied to a sharp tip with a curvature radius $r_{w}$, located at some distance L from the ground plate perpendicular to it. The electric field generated by corona discharge in the space between the electrodes as a function of the distance *x* from the needle tip can be expressed as in Equation (2). In this case, the curvature radius $r_{w}$ is approximated with the needle half diameter d=0.35 mm and the electric field is evaluated at the distance x=L from the sample surface, variable from 1 cm to 5 cm.

These measurements were completed as a calibration of the experimental setup to have a reference for the following ones, but also to obtain the optimal distance and respective voltage for a maximum sample charging, the reason being that, in a corona discharge phenomenon, electrons scattered by air atoms (O${}_{2}$ and N${}_{2}$) and the same atoms once ionized are accelerated by electric field toward sample surface up to penetrate on outer layers [36]. Therefore, by varying the distance between the negatively charged needle and the positively charged sample surface on the ground plate, the surface charge density is changing. For large distances, more ions can reach the sample compared to electrons scattered many times on a long path, while for small distances more electrons can reach the sample on a short path compared to ions. The optimal distance for a maximum charging is found to be 4 cm with a generator voltage value of −25 kV after potential measurements on samples surface through the electrometer.

With this optimal distance, the same LW-PLA samples were polarized for 5 min at −25 kV, at different temperatures from 80 °C to 110 °C with steps of 10 °C, to find an optimal pre-heating temperature. In this case, the disks were placed on a heated plate. Once the temperature was set, we waited for the heat to be transmitted throughout the entire sample. The waiting time was calculated following the Newton law of cooling/heating:

(3) $$T\left(t\right)=T_{S}+\left(T_{0}-T_{S}\right)\cdot e^{-\frac{t}{\tau }},$$

where $T_{0}$ and $T_{S}$ are, respectively, the initial and final temperatures (if $T_{0}>T_{S}$, it describes cooling, while if $T_{0}<T_{S}$, heating) and the time constant

(4) $$\tau =\frac{mcd}{\lambda A},$$

depends on the sample mass, *m*, thickness, *d*, base area, *A*, PLA specific heat, c=1800 J/kg·K and thermal conductivity, λ=0.13 W/m·K [37]. After heating the sample for 50 s, corresponding to about 10 τ, corona discharge was activated. After 30 s, the heating was switched off and the corona discharge was kept active until the material cooled down to room temperature.

This procedure was adopted because when the polymer is brought back down its $T_{g}$, its chains are immobilized and the space charges are trapped. In this way, when the field is shut down, they do not respond to it, making the instant polarization disappear [37]. The cooling process of the samples was accelerated by placing cold water on the heated plate. The optimal pre-heating temperature is found to be 85 °C, having obtained with it in these measurements the highest potential values and being in the range between the glass transition and cold crystallization temperatures of the material. This sweet point of pre-heating temperature lies between the glass transition and melting point, as shown by the DSC curves analysis. In fact, in this temperature interval, the crystalline structures inside the material are also free to re-orientate due to the external electric field.

**Figure 10:**
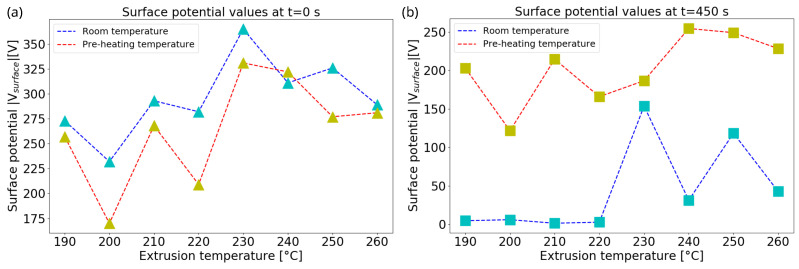
Maximum surface potential values obtained at *t* = 0 s (**a**) and *t* = 450 s (**b**), as a function of extrusion temperature, for samples polarized with corona discharge at RT and at *T* = 85 °C.

Once the pre-heating temperature is found, LW-PLA samples with different degrees of foaming were polarized at 4 cm and −25 kV for 5 min at RT and at 85 °C to investigate how temperature affects their charging efficiency. The maximum surface potential values and their temporal decay were measured under the electrometer for these two temperature configurations. As shown in Figure 10a, it is clear that the majority of maximum potential values (taken at t=0 s) for the thermally treated samples at 85 °C during charging tend to decrease in modulus by 70 V, while two of them printed at higher temperatures remain quite the same. This effect is due to the fact that, when the material temperature rises, the number of active trapping levels contributing to the charge movement increases, while the number of inactive trapping levels contributing to the charge storage decreases, leading to a lower value of the sample surface potential [38]. Nevertheless, the situation at longer times is very different (t=450 s), as displayed in Figure 10b, where the surface potentials of the thermally treated samples retain most of their values compared to those charged at RT. Despite this, some of the samples printed at temperatures above 230 °C and polarized at room temperature show a higher charge retention from those printed at lower temperature, probably due to the increasing foaming effect, i.e., the increasing presence of bubbles, starting from the optimal foaming temperature.

**Figure 11:**
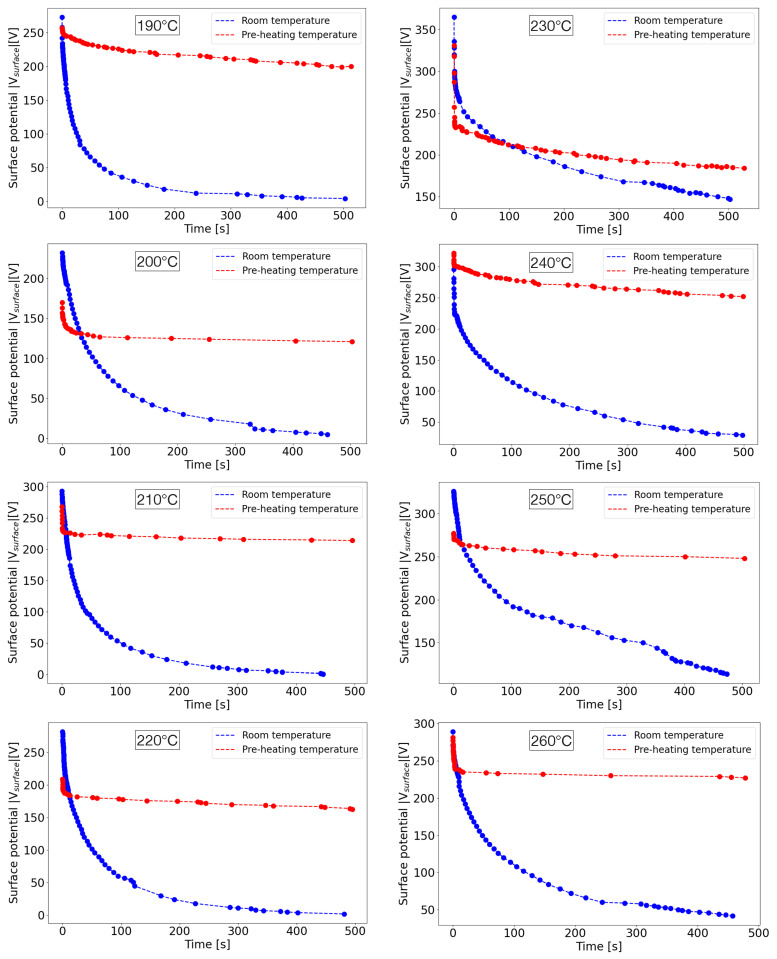
Temporal decay of surface potential values for the different degrees of foamed samples at RT and at *T* = 85 °C. Dots represent experimental data while dashed lines are guide for the eyes.

Measuring the potential values of thermally treated samples up to 500 s, as illustrated in Figure 11, it has been found that their temporal stability can be significantly improved in their decay. For some samples polarized at RT, it can be observed that the potential decay is fast and the value is very quickly reaching few volts, practically nullifying the induced charging by corona discharge. On the other hand, for samples polarized at 85 °C, the decay of the potential is fast only within the first seconds but then the values remain stable up to 8 min for almost the totality of extrusion temperatures.

**Figure 12:**
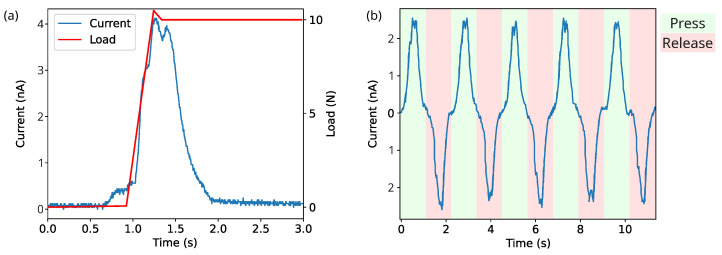
(**a**) Short-circuit current and applied force when the sample is under compression. (**b**) Short-circuit current in a series of compression and release cycles.

With regard to the piezoelectric activity of the 3D-printed samples, Figure 12a shows the temporal behaviour of the short-circuit current measured on a charged sample of 0.5 mm of thickness extruded at 240 °C when it is subjected to a step-like compression force up to *F* = 10 N. By integrating this curve, the total generated charge *Q* is obtained. Thereby, the quasi-static piezoelectric $d_{33}$ coefficient is determined by

(5) $$d_{33}=\frac{Q}{F}=\frac{\sigma_{s}}{P},$$

where $\sigma_{s}$ is the surface charge density and *P* is the applied stress. According to this test, the obtained value of effective $d_{33}$ coefficient is 212 pC/N. This value is in agreement with those obtained in previous works; for example, Zhukov et al. [13] obtained quasi-static piezoelectric coefficients of foamed PLA between 100 and 600 pC/N and 44 pC/N for $d_{31}$, while Vadas et al. [15] achieved values between 50 and 320 pC/N through a CO${}_{2}$ assisted extrusion foaming process. Figure 12b shows the generated short-circuit current along a series of compression and release cycles between 2.5 and 10 N. As expected, the current reverses during the release phase and it can be noted that the maximum and minimum values remain stable.

## 4. Conclusions

In this work, 3D-printable foamed commercial PLA was investigated as cellular piezoelectret material. Along with morphological, thermal and mechanical analysis, our study focused on the optimal 3D printing parameters in order to obtain the highest performance in terms of charge storage and piezoelectret performance. In particular, disk-shaped samples were thermally treated during negative corona discharge polarization. The results indicated optimal extrusion temperatures of 230 °C and 240 °C with extrusion flowrates of 53% and 44%, respectively. Subsequently, the PLA samples that were heated at the temperature of 85 °C under corona charging showed the most stable potential below −250 V, corresponding to the extrusion temperature of 240 °C. The material porosity reached its maximum of 77% at 240 °C, at which point the cell shape showed to be rounded, thus confirming the optimal foaming temperature. By increasing the temperature over 240 °C, the asphericity and squeezing of the cells increased and the charge stability started to degrade. Moreover, the samples charged at room temperature and extruded over 240 °C showed a stable potential up to 150 V, although instability in the measurements was encountered. These samples demonstrate a stable piezoelectric effect under a series of compress and release cycles with an effective $d_{33}$ coefficient of 212 pC/N, which is comparable with the values obtained in previous works on cellular PLA [13,15] and PP [7,39].

This study shows the possibility to implement foamed PLA piezoelectret in a double-step process: 3D printing and charging. The results demonstrate the potentiality to design a bio-compatible and sustainable piezoelectric device for energy-harvesting and bio-sensing applications. In perspective, this study suggests further development of 3D-printed piezoelectret devices based on foamed PLA with more complex architectures, such as beams array, springs, meta-materials and auxetic structures with integrated lead-free piezoelectric nanoparticles or conductive materials.

## Figures and Tables

**Table 1:** Flowrate values as a function of the nozzle extrusion temperature [20].

Temp	190 °C	200 °C	210 °C	220 °C	230 °C	240 °C	250 °C	260 °C
Flowrate	100%	100%	100%	85%	53%	44%	44%	48%

**Table 2:** Average sample thickness d and cell density σ as a function of the extrusion temperature.

Temp	190 °C	200 °C	205 °C	210 °C	220 °C	230 °C	240 °C	250 °C	260 °C
d (μm)	576.7	570.2	606.4	604.3	642.1	665.5	638.5	562.1	556.5
$\sigma \left(\frac{\mathrm{cell}}{{\mathrm{mm}}^{2}}\right)$	/	/	74.2	109.7	221.2	238.2	227.4	213.8	217.9

**Table 3:** Models for the Young’s modulus of porous materials and fitting results.

Reference	Model	Adjustable Parameters
Gibson and Ashby [32]	$E_{s}\left(\phi^{2}{\left(\frac{\rho_{\mathrm{cellular}}}{\rho_{\mathrm{solid}}}\right)}^{2}+\left(1-\phi \right)\frac{\rho_{\mathrm{cellular}}}{\rho_{\mathrm{solid}}}\right)$	ϕ=0.95±0.10
Ramakrishnan and Arunachalam [33]	$E_{s}\left[\frac{{\left(\rho_{\mathrm{cellular}}/\rho_{\mathrm{solid}}\right)}^{2}}{1+\left(2-3\nu_{0}\right)\left(1-\rho_{\mathrm{cellular}}/\rho_{\mathrm{solid}}\right)}\right]$	$\nu_{0}=0.36$
Arnold et al. [34]	$E_{s}{\left(\rho_{\mathrm{cellular}}/\rho_{\mathrm{solid}}\right)}^{1.21\cdot {\left(a/b\right)}^{1/3}}$	a/b=7.0±1.5

**Table 4:** Needle-sample surface distances, generator voltage and calculated electric field values.

L (cm)	1	2	3	3.5	4	4.25	4.5	4.75	5
*V* (kV)	−6	−8	−18	−20	−25	−25	−25	−25	−25
*E* ($\frac{\mathrm{kV}}{\mathrm{cm}}$)	1.45	0.84	1.16	1.07	1.15	1.07	1.00	0.94	0.88

## Data Availability

Data are contained within the article.
